# The Ethics of Switch/Simplify in Antiretroviral Trials: Non-Inferior or Just Inferior?

**DOI:** 10.1371/journal.pmed.1001240

**Published:** 2012-07-17

**Authors:** Andrew Carr, Jennifer Hoy, Anton Pozniak

**Affiliations:** 1 Clinical Research Program, Centre for Applied Medical Research, St Vincent's Hospital, Sydney, Australia; 2 HIV/Immunology/Infectious Diseases Unit, St Vincent's Hospital, Sydney, Australia; 3 Infectious Diseases Unit, Alfred Hospital, Melbourne, Australia; 4 Department of Infectious Diseases, Faculty of Medicine, Nursing and Health Sciences, Monash University, Melbourne, Australia; 5 Chelsea and Westminster Hospital, London, United Kingdom

## Abstract

Using the SWITCHMRK and MONET trials as examples, Andrew Carr and colleagues question the ethics and motives of switch or simplification trials of anti-retroviral therapy.

**Summary Points**

- The high efficacy of antiretroviral therapy has resulted in more trials that switch or simplify existing therapy in patients whose HIV is fully controlled.
- The primary outcome of about half of these trials is virological non-inferiority. As participants already have fully controlled HIV on existing therapy, these trials offer no virological benefit.
- Many trials (i) enrol patients who cannot benefit with the switch, (ii) do not capture (or report) all potential risks, and (iii) are designed with a view to a pharmaceutical company's profits rather than participant benefit.
- A switch/simplification trial is only ethical if participants can meaningfully benefit from the treatment change and are more likely to benefit than suffer harm, and if the study is powered to assess the key expected benefit and reports all end points.

## Introduction

The efficacy of new antiretroviral therapy (ART) drugs is established in phase 3 trials, including in patients with virological failure (i.e., ongoing viral replication) on existing ART. In these trials, a new drug generally demonstrates improved virological control relative to placebo. Improved ART efficacy over the last 15 years has led to a dramatic decrease in the numbers of treatment-experienced patients with virological failure [1]. Because fewer patients are failing ART in resource-rich settings, enrolling such patients in superiority trials of newer drugs has become increasingly difficult.

One response to fewer patients with virological failure is to evaluate new ART drugs in patients receiving effective ART, with undetectable plasma HIV. One of us (AC) participated in one such trial that yielded adverse patient outcomes, and another (AP) independently collated these trials in the process of developing an educational resource.

## Switching and Simplifying Antiretroviral Therapy in Clinical Trials

There are two types of ART efficacy studies performed in patients receiving effective ART and with undetectable plasma HIV. More commonly, one ART drug is switched to a new drug under development, the primary end point being virological non-inferiority, i.e., that virological suppression can be maintained to a similar degree as with current ART. Secondary end points such as quality of life, treatment simplicity, and toxicity are often of greater interest, provided greater virological failure does not occur with the new ART drug.

A similar virological non-inferiority trial involves ART simplification, which takes one of two forms. A new co-formulation can replace the same two or three drugs taken separately, with the aim of reducing pill burden and possibly improving treatment adherence and cost. Alternatively, one existing drug is ceased without the introduction of a new drug.

The primary aim of a non-inferiority trial is to show that a new treatment is about as effective as existing therapy for the primary outcome (with a small pre-specified between-group difference and 95% confidence interval for that difference). In ART switch studies, the investigators typically hope to be able to state with 95% confidence that new drug B is no more than 10%–12% worse than existing drug A at controlling HIV replication. Because of the similar outcomes expected, non-inferiority trials are less likely than superiority trials to improve patient health, and greater attention should be placed on other potential advantages and disadvantages (secondary outcomes).

There are several potential advantages and disadvantages for patients of switching or simplifying ART (Table 1). Potential advantages include reduced toxicity, pill burden, or cost. One key potential disadvantage is that effective, well-tolerated ART is abandoned. This is likely to be of increasing importance given the diminishing new HIV drug pipeline.

**Table 1:** Switching and simplifying antiretroviral therapy in a patient with controlled HIV replication.

Treatment Aspect	Potential Advantages of Switching or Simplification	Potential Disadvantages of Switching or Simplification
**Efficacy**	Improved drug levels may improve efficacy	Loss of virological control
**Pill burden**	Reduce doses per day, tablets per day or meal restrictions (improve quality of life)	Increase pill doses or number
**Toxicity**	Prevent or reverse toxicity	Toxicity of new drug may be greater than toxicity of existing drug (new drugs have less long-term safety data than older drugs)
		Toxicity may not reverse
		Switching may be less effective than other approaches, e.g., statins for hypercholesterolaemia, smoking cessation for cardiovascular risk
**Drug interactions**	Prevent or reduce drug interactions	Unforeseen new interaction
**Co-morbid disease**	Prevent or reduce co-morbidities	Adverse interaction, e.g., lipid increase in patient with cardiovascular disease
**Pregnancy**	Prevent toxicity to mother or foetus	New toxicity to mother or foetus
**Costs**	Reduce costs for patient or improve community coverage with same health-care expenditure	Increase costs because of greater virological failure, toxicity with new therapy
		Future market prices may change
**Confidentiality**	Improve confidentiality by not requiring pill refrigeration or dosing at work	
**Treatment options**	Enable use of a drug previously avoided because concerns about medication safety or efficacy no longer apply	Reduce future options—the number of new HIV drugs in clinical development is small and reducing
**Pharmacy**	Lower pharmacy costs	Patient takes the wrong dose or pills
		Pharmacy prescribes the wrong agent
		Forgotten drug interactions or superimposed toxicities

Two important virological findings have emerged from HIV switch and simplification studies. First, neither type of study reduces the risk of virological failure. Indeed, simplification with protease inhibitor monotherapy can increase this risk [2]. Second, non-inferior results in a switch trial have sometimes created the incorrect perception that these investigational regimens are as potent as more proven regimens, only to be found inferior when formally evaluated in patients initiating ART for the first time [3]–[6].

## Risks and Benefits for Clinical Trial Participants

The Declaration of Helsinki summarises the World Medical Association's perspective on the risks and benefits of clinical trials (Box 1) [7]. The benefits of any intervention should outweigh the perceived potential risks, and the health of the individual patient should generally take precedence over the potential public good.

**Box 1. Text from the 2008 Declaration of Helsinki Relating to Potential Risks and Benefits of Human Research**

- “In medical practice and in medical research, most interventions involve risks and burdens."
- “Every medical research study involving human subjects must be preceded by careful assessment of predictable risks and burdens to the individuals and communities involved in the research in comparison with foreseeable benefits to them and to other individuals or communities affected by the condition under investigation."
- “Physicians may not participate in a research study involving human subjects unless they are confident that the risks involved have been adequately assessed and can be satisfactorily managed. Physicians must immediately stop a study when the risks are found to outweigh the potential benefits or when there is conclusive proof of positive and beneficial results."
- “Medical research involving human subjects may only be conducted if the importance of the objective outweighs the inherent risks and burdens to the research subjects."
- “[E]ach potential subject must be adequately informed of the aims, methods, sources of funding, any possible conflicts of interest, institutional affiliations of the researcher, the anticipated benefits and potential risks of the study."
- “For medical research combined with medical care…the benefits, risks, burdens and effectiveness of a new intervention must be tested against those of the best current proven intervention."

A switch or simplification study, however, with a primary end point of continued virological suppression and with no clinically useful impact on toxicity, costs, or quality of life cannot have any benefit to the participant. And yet, of the 96 HIV switch or simplification trials registered at ClinicalTrials.gov (as of November 24, 2011), 44 (46%) trials had virological non-inferiority as the primary end point. In many studies, non-virological end points are not reported and sometimes not included in data collection.

Disadvantages of existing ART, with respect to toxicity, quality of life, cost, or other issues, can only be addressed in switch or simplification studies if several key issues are handled. First, a particular disadvantage (typically toxicity) of current therapy must be a key and well-defined entry criterion, with recruitment of sufficient numbers of at-risk participants to provide statistical power to yield clinically meaningful results. Good examples of this approach are trials that evaluated the ability of drug switching to reverse objectively defined lipoatrophy and efavirenz-related central nervous symptoms [8],[9].

Second, the relevant disadvantage(s) of the switch should be measured, analysed, and reported. Participants must be informed about all potential advantages and disadvantages and which is the primary focus of the study. It is not sufficient to state “you may not receive any benefit" in an informed consent form; “we anticipate the risks and benefits to be…" would be more transparent. If cost is the main focus, the consent form should state “we do not anticipate any clinical benefit for you, only a cost benefit, and the potential risks to you are…".

Lastly, some laboratory parameters commonly reported as key positive outcomes of switch or simplification trials may not represent significant toxicities. For example, switching ART to improve dyslipidaemia can only be beneficial in individuals for whom cardiovascular risk reduction is clinically indicated. Generally, these would be patients whose overall cardiovascular risk is elevated (e.g., ≥10% risk of myocardial infarction over 10 years using the Framingham equation).

Of the HIV switch or simplification trials at ClinicalTrials.gov, 52 (54%) had a non-virological primary end point, most commonly relating to pharmacokinetics (*n* = 11), lipids (*n* = 7), cardiovascular function (*n* = 6), or HIV lipoatrophy (*n* = 5). In terms of relevance, no lipid switch study specifically enrolled patients with elevated cardiovascular risk, whereas all lipoatrophy studies enrolled patients with some degree of lipoatrophy.

## The SWITCHMRK Switch Trials

The SWITCHMRK trials illustrate several limitations of ART switch trials. The two SWITCHMRK trials were phase 3, double-blind studies designed “to investigate the potential of substituting raltegravir (an HIV integrase inhibitor) for ritonavir-boosted lopinavir (an HIV protease inhibitor) to improve the adverse event profile of lopinavir-ritonavir-based combination regimens in stable HIV-infected patients who had achieved viral suppression" [10].

The SWITCHMRK design was suboptimal. Patients with prior treatment failure were eligible, thus permitting some patients with pre-existing antiretroviral resistance to change to what was effectively raltegravir monotherapy; about 5% of patients in each study who switched to raltegravir rapidly experienced virological failure. The entry criteria guaranteed no clinical benefit for most participants. In addition, no overall toxicity benefit was demonstrated. The baseline prevalence of diarrhoea (a well-recognised side effect of lopinavir) was not reported, so it is not known whether diarrhoea improved with raltegravir, although raltegravir recipients had less grade 3 or 4 diarrhoea than patients continuing on lopinavir (3% versus 1%). The impact of the lower diarrhoea incidence on quality of life was not reported, nor was it determined what number of lopinavir recipients should be recruited to demonstrate any particular benefit.

Although serum cholesterol levels significantly declined with raltegravir, patients were not required to have an elevated cardiovascular risk at baseline, and the total cholesterol to high-density lipoprotein cholesterol ratio did not decline. For both these reasons, it is unlikely that the cholesterol reductions observed were clinically significant: there was no reduction in the number of cardiovascular events (unlikely given the sample size), and atherosclerosis burden was not measured (e.g., by carotid ultrasound).

Lastly, after the study was performed, raltegravir was substantially more expensive than lopinavir; no cost-effectiveness analysis was reported.

## The MONET Simplification Study

The MONET trial is an example of a simplification study with limitations. In MONET, adults with undetectable HIV viral load on ART including two nucleoside analogue reverse transcriptase inhibitors were randomised to switch ART to two nucleoside analogues plus ritonavir-boosted darunavir (a protease inhibitor), or to simplify ART by taking only ritonavir-boosted darunavir monotherapy [11]. Reduced toxicity, improved quality of life, and reduced health-care costs were all cited in the MONET publication as potential benefits of the trial's strategies, but none of these parameters was reported.

Although drug costs were lower with darunavir monotherapy, these reduced drug costs might have been offset by the additional clinical and laboratory costs associated with the higher rate of low-level viraemia in the simplification group. No cost-effectiveness analysis was reported.

## Elephants in the Room

If there is little gain from most HIV switch and simplification studies with virological primary end points, why do so many occur? We believe most patient information and consent forms do not explain all potential risks and benefits or the risk–benefit balance, and that patients participate because they trust their treating doctor, who in turn might be motivated by the opportunity to use new therapy, by academic acclaim, and/or by remuneration. Market penetration is another pharmaceutical company motivation.

If the science underlying such trials is often so tenuous, why do ethics committees continue to approve their conduct? We believe the many committees are not sufficiently attuned to the abovementioned risks, benefits, incentives, and motivations, a problem that will increase with the trend for multi-site approval by a single ethics committee.

## Switching and Simplifying Therapy in Future Clinical Trials

Virological non-inferiority is essential in switch and simplification studies, but should not be the only end point, as virological non-inferiority in isolation is not a benefit. When reviewing such trials, ethics committees should approve studies only if they are convinced the potential gain is both clearly anticipated and clinically relevant to all participants, and confident that participants are not likely to be at risk of virological failure. Non-inferiority switch or simplification trials that primarily benefit a pharmaceutical company should not be contemplated or approved. These studies must demonstrate the potential for clinically meaningful benefits in patients that need them, i.e., in those who have a toxicity or are at high risk, and where the toxicity is clinically meaningful, not just an abnormal laboratory parameter. The switch should be double-blind for subjective end points. Large switch toxicity studies should be preceded by small pilot studies to demonstrate proof of concept. The virological potency of ART is best assessed in trials in ART-naïve adults, not in switch studies.

Switch and simplification studies should not be so long as to disadvantage a control group, and only for as long as would be expected for a given toxicity to improve. Lipid switch studies probably need last only 12 weeks, whereas bone mineral density studies would need to last at least 12 months. An immediate versus delayed switch design is likely to be more attractive to patients.

Switch and simplified maintenance studies may benefit from quality of life, cost-effectiveness, and number-needed-to-treat-to-benefit analyses. Studies whose primary aim is reduced costs and that have no benefit to the patient are acceptable provided patients are clearly informed of this primary aim and that the cost benefits are reported.

Perhaps the International Committee of Medical Journal Editors and independent medical journals should mandate submission of patient information and consent forms with submitted manuscripts to determine whether patients were fully informed about the risks and benefits of a trial, all risks and benefits were reported, and the principles of the Declaration of Helsinki were upheld. Approved patient information and consent forms should be posted on committee and/or sponsor websites.

## Switching and Simplifying Therapy in Clinical Practice

Switching ART regimens when virologically suppressed may be appropriate for many reasons, but the full risk–benefit profile should be determined beforehand. The diminishing antiretroviral drug pipeline suggests greater care will need to be given in coming years to extending the benefits of existing drugs for what is likely to remain lifelong therapy.

## Abbreviations

ART: antiretroviral therapy
